# Aflatoxin and viral hepatitis exposures in Guatemala: Molecular biomarkers reveal a unique profile of risk factors in a region of high liver cancer incidence

**DOI:** 10.1371/journal.pone.0189255

**Published:** 2017-12-13

**Authors:** Joshua W. Smith, Maria F. Kroker-Lobos, Mariana Lazo, Alvaro Rivera-Andrade, Patricia A. Egner, Heiner Wedemeyer, Olga Torres, Neal D. Freedman, Katherine A. McGlynn, Eliseo Guallar, John D. Groopman, Manuel Ramirez-Zea

**Affiliations:** 1 Department of Environmental Health and Engineering, Bloomberg School of Public Health, Johns Hopkins University, Baltimore, MD, United States of America; 2 Research Center for the Prevention of Chronic Diseases, Institute of Nutrition of Central America and Panama, Guatemala City, Guatemala; 3 Department of Epidemiology, Bloomberg School of Public Health, Johns Hopkins University, Baltimore, MD, United States of America; 4 Department of Medicine, School of Medicine, Johns Hopkins University, Baltimore, MD, United States of America; 5 Department of Gastroenterology, Hepatology, and Endocrinology, Hannover Medical School, Hannover, Germany; 6 Laboratorio Diagnóstico Molecular, Guatemala City, Guatemala; 7 Division of Cancer Epidemiology and Genetics, National Cancer Institute, NIH, Rockville, MD, United States of America; Centre de Recherche en Cancerologie de Lyon, FRANCE

## Abstract

Liver cancer is an emerging global health issue, with rising incidence in both the United States and the economically developing world. Although Guatemala experiences the highest rates of this disease in the Western hemisphere and a unique 1:1 distribution in men and women, few studies have focused on this population. Thus, we determined the prevalence and correlates of aflatoxin B_1_ (AFB_1_) exposure and hepatitis virus infection in Guatemalan adults. Healthy men and women aged ≥40 years (n = 461), residing in five departments of Guatemala, were enrolled in a cross-sectional study from May—October of 2016. Serum AFB_1_-albumin adducts were quantified using isotope dilution mass spectrometry. Multivariate linear regression was used to assess relationships between AFB_1_-albumin adduct levels and demographic factors. Biomarkers of hepatitis B virus and hepatitis C virus infection were assessed by immunoassay and analyzed by Fisher’s exact test. AFB_1_-albumin adducts were detected in 100% of participants, with a median of 8.4 pg/mg albumin (range, 0.2–814.8). Exposure was significantly higher (p<0.05) in male, rural, low-income, and less-educated participants than in female, urban, and higher socioeconomic status participants. Hepatitis B and C seropositivity was low (0.9% and 0.5%, respectively). Substantial AFB_1_ exposure exists in Guatemalan adults, concurrent with low prevalence of hepatitis virus seropositivity. Quantitatively, AFB_1_ exposures are similar to those previously found to increase risk for liver cancer in Asia and Africa. Mitigation of AFB_1_ exposure may reduce liver cancer incidence and mortality in Guatemala, warranting further investigation.

## Introduction

The increasing burden of cancer morbidity and mortality across diverse populations has emerged as a major global health concern for this century [1]. Primary liver cancer (LC) is emblematic of these global challenges. It is a common cancer, with nearly 800,000 cases and over 750,000 deaths worldwide in 2012 –fifth and second, respectively, among all cancer sites [2]. The burden of this highly lethal disease is not borne evenly, as LC occurs with a disproportionately high incidence in the economically developing world, where over 80% of new cases occur [3,4]. Although LC incidence and mortality still remain the highest in historically afflicted regions (China, southeastern Asia, sub-Saharan Africa) [5], global trends have begun to shift towards traditionally lower-risk countries. For example, while rates in China, the Philippines, and Singapore all decreased from 1993–2007, incidence in the United States, the United Kingdom, Canada, Australia, Germany, and Switzerland has risen [6]. In the United States, although death from most cancer types has been in decline, LC mortality increased between 2003–2012 at the highest rate of any organ site [7]. Such changes likely reflect shifts in factors that influence LC risk. LC is one of the few tumor types for which major etiological risk factors have been identified, which include chronic infection with hepatitis B virus (HBV) or hepatitis C virus (HCV), excessive alcohol consumption, obesity, nonalcoholic fatty liver disease and exposure to the foodborne mycotoxin, aflatoxin [2,8]. In China and Taiwan, vaccination against HBV coupled with reduced exposure to aflatoxin led to the declines in LC incidence seen in those populations [9–11], while obesity has likely contributed to the rise in LC in the U.S. [12].

Although LC etiology in Asian and African regions has been under constant investigation for decades and countries such as the U.S. have experienced rapid investments in research to combat increasing LC incidence, others have been left adrift amidst a rising epidemic. Guatemala, with an estimated age-standardized incidence of 16.0 per 100,000, is one such country. In fact, Guatemala suffers from the highest liver cancer incidence rate in the Western hemisphere, nearly three times that of the U.S. [4,5]. Put in context, this is a higher age-standardized rate than Japan, the Philippines, or Singapore, and only 30% lower than China. To our knowledge, no studies have investigated etiological factors contributing to the high rates of LC in Guatemala. Moreover, the distribution of LC *within* the Guatemalan population is unique–while in the vast majority of countries men have two- to three-fold higher rates of both LC incidence and death than women [6], in contrast, ratios in Guatemala are 1:1 [5]. Other countries in the region, such as El Salvador and Nicaragua, have similarly uncommon distributions, although lower rates of LC. The conventional disparity in LC incidence between sexes is commonly suggested to be the result of higher prevalence of LC risk factors in men than in women, but could be multifactorial and is not completely understood. Needless to say, given the dearth of work on LC in Guatemala, the factors driving the unusual distribution of LC incidence within this country is unclear. The high burden of LC, combined with its uncommon sex distribution, reveals a pressing need for the examination of risk factors driving this disease in Guatemala and Central America.

Aflatoxins are produced by molds of the genus *Aspergillus*–namely *A*. *parasiticus* and *A*. *flavus* [13,14]–and exposure occurs through consumption of contaminated commodity grains, particularly maize [13,14]. Aflatoxin B_1_ (AFB_1_) is the most toxic of the aflatoxins, and, since 1993, has been designated a known human carcinogen by the International Agency for Research on Cancer (IARC) [15,16]. Due to the widespread distribution of *Aspergillus*, uncontrolled food contamination in developing countries, and the carcinogenic potency of aflatoxins, exposure to AFB_1_ may account for one quarter of global liver cancer burden [17,18]. Maize is consumed as a staple crop throughout Latin America, making aflatoxin-contaminated food a potentially insidious health hazard in these countries. Aflatoxin contamination in Guatemala has been assessed in a small number of food survey analyses [19,20], including a recent survey of maize that found AFB_1_ contamination in 21 of the 22 departments of Guatemala [21]. While they demonstrate widespread contamination by aflatoxins, these surveys are compromised by the high heterogeneity of aflatoxin levels within a bulk of contaminated food [14] and, more fundamentally, cannot be used to predict subject-level dose. In contrast, biomarkers of aflatoxin exposure have been validated as measures of internal and biologically effective dose [13], and their epidemiological use has proven crucial to the establishment of aflatoxins as etiological agents of primary liver cancer [13,15,16,22,23]. In particular, the AFB_1_-serum albumin adduct [24,25] is useful not only for estimation of liver cancer risk [26], but also–thanks to the long half-life of albumin in circulation [27]–for monitoring past exposures on a timescale of months.

No studies to date have utilized validated biomarkers to assess aflatoxin exposure in Guatemalans—searches of PubMed, EMBASE, Scopus, and Web of Science for (“aflatoxin” AND “Guatemala”) returned zero relevant articles published on or before June 23, 2017. Furthermore, since hepatitis viruses are major etiological factors for LC [12,28] and HBV infection interacts with aflatoxin exposure to greatly increase liver cancer risk [22,23], hepatitis virus exposures should be assessed in conjunction with aflatoxin. Thus, the goal of this cross-sectional study was to assess the prevalence of aflatoxin, HBV and HCV exposure in Guatemalan adults, in order to better understand factors potentially driving this country’s high incidence of LC.

## Methods

### Subjects and study design

**Fig 1:**
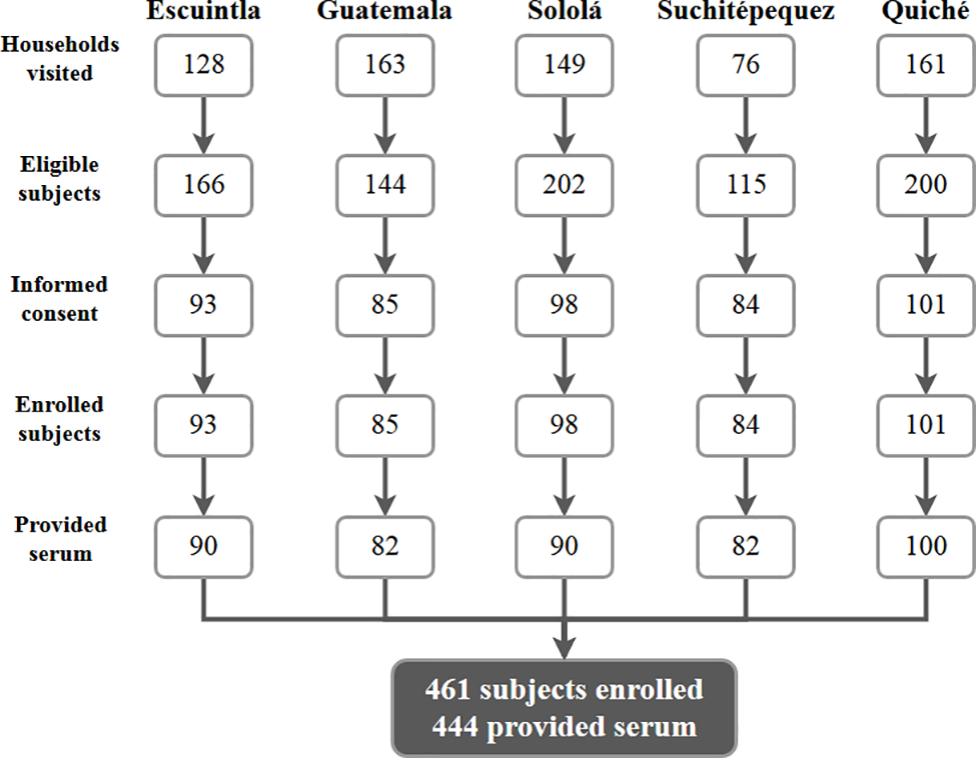
Flow-chart of recruitment. Numbers of households or participants at each stage of recruitment, for each department and for the final sample.

All participants provided written informed consent. All study procedures were approved by Institutional Review Boards at Johns Hopkins Bloomberg School of Public Health and the Institute of Nutrition of Central America and Panama (INCAP). Guatemalan men and women ≥ 40 years of age were recruited from cities in five Guatemalan departments: Chichicastenango (Quiché department), Escuintla (Escuintla), Mixco (Guatemala), San Lucas Tolimán (Sololá), and San Pablo Jocopilas (Suchitépequez). These cities were selected to represent urban (Escuintla, Mixco) and rural settings (Chichicastenango, San Lucas Tolimán, and San Pablo Jocopilas) throughout the country. Participants were recruited and enrolled between May and October of 2016. Maps of each community, when available, were used to pre-select households. Households were visited and a maximum of two non-blood-related participants per household were invited to participate in the study. We excluded pregnant women and individuals who could not provide informed consent. All field procedures were conducted by experienced, trained personnel. Demographic information was obtained during the initial home visit by study personnel-administered questionnaire. Ethnic background (indigenous or non-indigenous) was determined by participants’ self-identification. Serum samples were collected during a subsequent clinic visit; most samples were collected less than a week after the initial home visit. We enrolled a median of 93 participants from each locality (range, 84–101). In total, we contacted 677 households with 827 eligible individuals and enrolled 461 participants (56% response rate) from 358 households; 444 provided serum samples (**Fig 1**).

### Aflatoxin B_1_ albumin adduct measurement and hepatitis B and C viral assessment

AFB_1_ exposure was assessed with minor variations to the method reported by McCoy *et al*. [29]. 443 of 444 serum samples collected had suitable volume for AFB_1_ analysis. Serum (250 μL) was spiked with an internal standard (0.5 ng AFB_1_-d4-lysine in 100 μL), combined with Pronase (EMD Millipore, Billerica MA, USA) protease solution (3.25 mg in 0.5 mL phosphate-buffered saline), and incubated for 18 h at 37°C. Solid-phase extraction–processed samples (Oasis MAX columns; Waters, Milford, MA, USA) were analyzed with isotope dilution mass spectrometry on a ThermoFisher Scientific TSQ Vantage (Waltham, MA, USA) in positive electrospray ionization mode. Peaks were manually integrated and AFB_1_-albumin lysine adducts (AFB_1_-lys) were normalized to total serum albumin as pg AFB_1_-lys/mg albumin. Serum total albumin was measured spectrophotometrically by ELISA, with minor modifications to manufacturer’s instructions (ab179887; Abcam, Cambridge, MA). The limit of quantification for this method was 0.2 pg AFB_1_-lys/mg albumin.

HBV infection status was assessed via determination of hepatitis B surface antigen (HBsAg) and total antibodies to hepatitis B core antigen (anti-HBc), while HCV status was assessed via testing for antibodies to hepatitis C virus (anti-HCV). All viral markers were assessed in the hepatitis diagnostic laboratory of Hannover Medical School as previously described [30].

### Statistical analyses

Stata version 14 (StataCorp, College Station, TX, USA) and SAS v9.4 (Cary, NC, USA) were used for statistical analyses. The distribution of AFB_1_-lys was right-skewed and values were log10-transformed before analysis. We used multivariate linear regression to test for differences in AFB_1_-lys geometric means between demographic sub-groups, as well as to calculate marginally adjusted AFB_1_-lys geometric means with adjustment for age (continuous), sex, residence (urban *vs*. rural), and ethnicity (indigenous *vs*. non-indigenous). Sub-group differences in viral marker positivity were determined by Fisher’s exact test. Results were considered significant at p < 0.05.

## Results

The sociodemographic characteristics of the study participants are depicted in **Table 1**. The mean age of the participants was 55.4 years overall– 58.0 years for men and 53.5 years for women. More women (n = 262, 58.6%) than men (n = 199, 43.2%) were recruited to the study. Over 60% of the participants came from rural communities and slightly more than half of participants (54.0%) self-identified as being of indigenous ancestry. In total, two-thirds of the study participants had 0–5 years of formal education and a similar fraction of the participants had a monthly income of 1,501–4,500 quetzales, which is approximately $200–600 USD.

**Table 1:** Sociodemographic characteristics of study participants.

	Total	Male	Female	p-value
	461 (100%)	199 (43.2%)	262 (58.6%)	
**Age**				0.001
40–49	163 (35.4%)	57 (28.7%)	106 (40.5%)	
50–59	147 (31.9%)	58 (29.2%)	89 (34%)	
60–69	100 (21.7%)	51 (25.6%)	49 (18.7%)	
70–79	41 (8.9%)	26 (13.1%)	15 (5.7%)	
≥ 80	10 (2.2%)	7 (3.5%)	3 (1.2%)	
**Residence**				0.87
Rural	283 (61.4%)	123 (61.8%)	160 (61.1%)	
Urban	178 (38.6%)	76 (38.2%)	102 (38.9%)	
**Indigenous**				0.92
Yes	249 (54.0%)	108 (54.3%)	141 (53.8%)	
**Education**				0.01
0–5 years	305 (66.2%)	119 (59.8%)	186 (71.0%)	
≥ 6 years	156 (33.8%)	80 (40.2%)	76 (29.0%)	
**Income**				0.83
0–1,500 Q/mo	107 (23.4%)	49 (24.8%)	58 (22.3%)	
1,501–4,500 Q/mo	314 (68.6%)	133 (67.2%)	181 (69.6%)	
4,501–7,500 Q/mo	37 (8.1%)	16 (8.1%)	21 (8.1%)	

Data are provided as n (%). Indigenous, self-identified as being of indigenous ethnic background. Q, Guatemalan quetzales (1 USD ≈ 7.5 Q).

**Fig 2:**
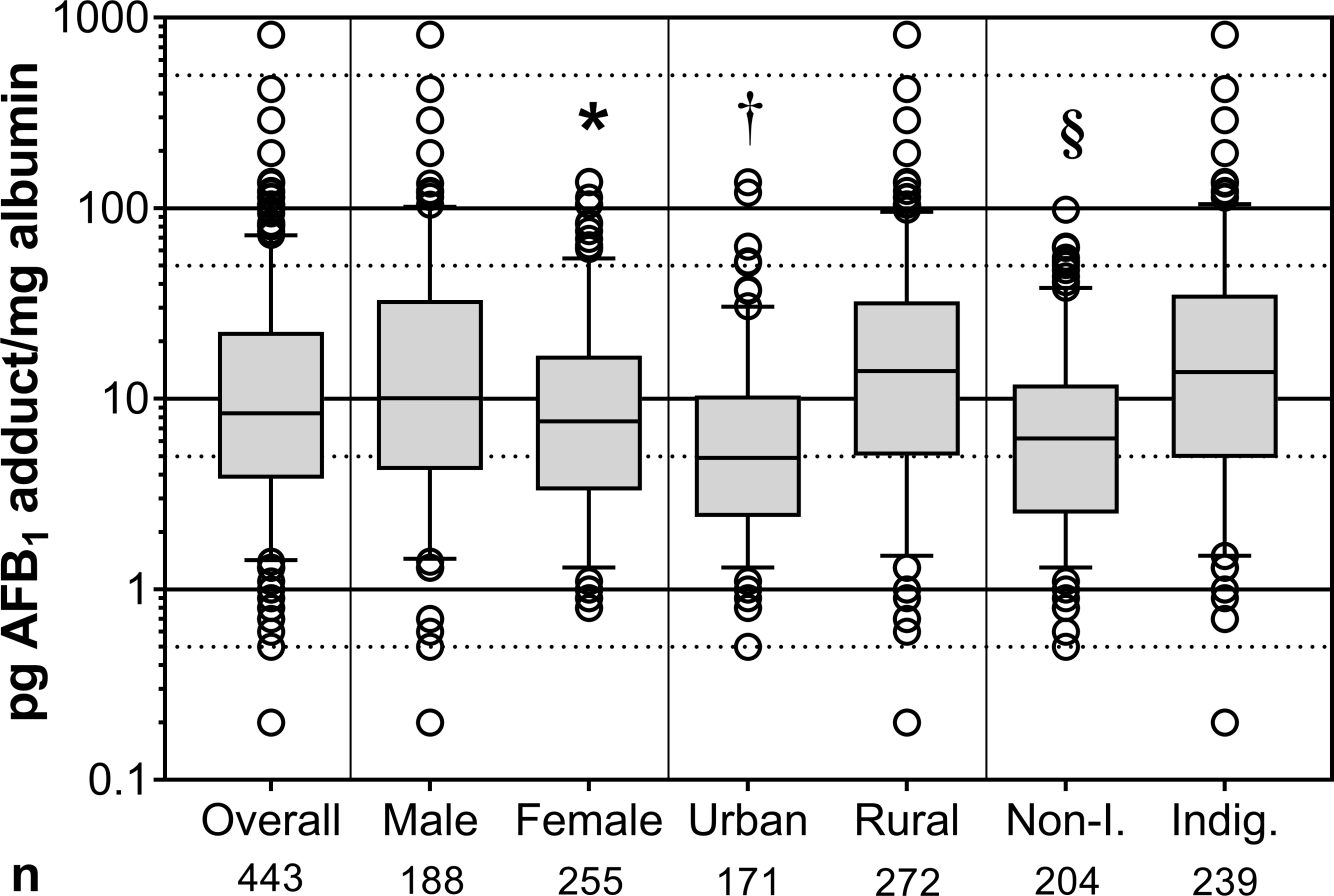
Unadjusted serum aflatoxin B_1_-albumin adduct levels (pg/mg) in study participants. Unadjusted serum AFB_1_-albumin adducts overall and with stratification by sex, residence location, or ethnicity. All data are plotted on a base-10 logarithmic scale; dotted scale lines are drawn at 0.05, 0.5, 5, 50, and 500 pg/mg. Whiskers on boxplots extend to the 5^th^ and 95^th^ percentiles, with individual observations beyond those cutoffs plotted. *, p = 0.009 *vs*. male; †, p ≤ 0.0001 *vs*. rural; §, p ≤ 0.0001 *vs*. indigenous by multiple linear regression of unadjusted adduct values. Sample size n is displayed below each group. Non-I., non-indigenous; Indig., indigenous.

Detectable levels of AFB_1_-lys were present in 100% of participants, with an overall range of 0.2–814.8 pg AFB_1_-lys/mg albumin and a median of 8.4 pg/mg. As seen in **Fig 2**and **Table 2**, stratification by demographic factors revealed several statistically significant differences among these sub-groups. Men had significantly higher circulating levels of AFB_1_-lys adducts than women. Adduct levels in rural participants were more than two-fold higher than in their urban counterparts, as were levels in participants who self-identified as being of indigenous ethnicity compared to those who self-identified as non-indigenous. We also observed significantly lower AFB_1_-lys levels in participants with ≥ 6 years of education compared to 0–5 years, and aflatoxin biomarker levels decreased significantly with increasing monthly income. AFB_1_ exposure was not associated with age (**Table 2**). Interestingly, we observed an interaction in unadjusted geometric means between residence location and self-identified ethnic status. While AFB_1_ levels were lower among non-indigenous participants in urban *vs*. rural areas (rural: 11.9 pg/mg; 95% CI 8.8, 15.9; urban: 4.9 pg/mg; 4.2, 5.8), the same was not true for indigenous participants (rural: 13.4 pg/mg; 11.2, 16.0; urban: 17.4 pg/mg; 8.3, 36.8).

**Table 2:** Unadjusted geometric means of serum aflatoxin-albumin adducts by sociodemographic characteristics of study participants.

	Geometric mean (95% CI) ^*a*^	Ratio of geometric mean (95% CI)	p-value for trend
**Age**			0.60
40–49	9.79 (8.16, 11.75)	1 (Reference)	
50–59	8.47 (6.90, 10.41)	0.87 (0.66, 1.14)	
60–69	9.34 (7.14, 12.21)	0.95 (0.69, 1.32)	
70–79	7.42 (4.83, 11.41)	0.76 (0.48, 1.21)	
≥ 80	12.32 (4.50, 33.73)	1.26 (0.47, 3.35)	
**Sex**			0.009
Male	10.93 (8.96, 13.34)	1 (Reference)	
Female	7.92 (6.89, 9.10)	0.72 (0.57, 0.92)	
**Residence**			≤ 0.0001
Rural	12.76 (10.94, 14.87)	1 (Reference)	
Urban	5.29 (4.56, 6.14)	0.41 (0.34, 0.51)	
**Indigenous**			≤ 0.0001
No	5.91 (5.14, 6.79)	1 (Reference)	
Yes	13.11 (11.08, 15.52)	2.22 (1.78, 2.76)	
**Education**			≤ 0.0001
0–5 years	11.02 (9.54, 12.73)	1 (Reference)	
≥ 6 years	6.18 (5.12, 7.44)	0.56 (0.44, 0.71)	
**Income**			≤ 0.0001
0–1,500 Q/mo	12.75 (10.23, 15.91)	1 (Reference)	
1,501–4,500 Q/mo	8.75 (7.56, 10.12)	0.69 (0.53, 0.89)	
4,501–7,500 Q/mo	4.64 (3.42, 6.29)	0.36 (0.25, 0.53)	

Indigenous, self-identified as being of indigenous ethnic background. Q, Guatemalan quetzales (1 USD ≈ 7.5 Q).

^*a*^ Values are pg AFB_1_-albumin / mg albumin.

Differences by residence location and ethnicity were even more pronounced when further stratified by sex, as rural and indigenous men experienced some of the highest exposures of any sub-group within our analyses. Levels in rural men (15.5 pg/mg; 95% CI: 11.9, 20.2) were more than two-fold greater than urban male residents (6.1 pg/mg; 95% CI: 4.7, 7.9), while men who identified themselves as indigenous were similarly exposed to rural men (17.2 pg/mg; 95% CI: 13.0, 22.7) and had AFB_1_ exposures more than two times higher than their non-indigenous male counterparts (6.4 pg/mg, 95% CI: 5.0, 8.1). Indigenous, rural males had the highest AFB_1_-lys albumin geometric mean adduct levels (16.7 pg/mg; 95% CI: 12.5, 22.2).

**Table 3**depicts AFB_1_-lys geometric means for each demographic subgroup, after simultaneous adjustment for all included demographic characteristics (age, sex, residence location, ethnicity, educational attainment, and income). After adjustment, only sex, residence location, education, and income remained significant predictors (p < 0.05) of AFB_1_-lys level.

**Table 3:** Adjusted geometric means of serum aflatoxin-albumin adducts by sociodemographic characteristics of study participants.

	Adjusted geometric mean (95% CI) ^*a*^^,^ ^*b*^	Ratio of adjusted geometric mean (95% CI)	p-value for trend
**Age**			0.13
40–49	9.97 (8.27, 12.01)	1 (Reference)	
50–59	8.89 (7.35, 10.75)	0.89 (0.69, 1.16)	
60–69	9.06 (7.18, 11.43)	0.91 (0.67, 1.24)	
70–79	6.18 (4.23, 9.04)	0.62 (0.39, 0.98)	
≥ 80	10.06 (4.88, 20.77)	1.01 (0.40, 2.58)	
**Sex**			0.001
Male	11.24 (9.49, 13.30)	1 (Reference)	
Female	7.69 (6.66, 8.89)	0.69 (0.55–0.86)	
**Residence**			≤ 0.0001
Rural	11.37 (9.62, 13.42)	1 (Reference)	
Urban	6.28 (5.00, 7.89)	0.56 (0.40, 0.77)	
**Indigenous**			0.24
No	8.16 (6.64, 10.04)	1 (Reference)	
Yes	9.87 (8.19, 11.90)	1.21 (0.87, 1.68)	
**Education**			0.003
0–5 years	10.22 (8.91, 11.73)	1 (Reference)	
≥ 6 years	7.07 (5.78, 8.65)	0.69 (0.54, 0.89)	
**Income**			0.01
0–1,500 Q/mo	10.77 (8.50, 13.65)	1 (Reference)	
1,501–4,500 Q/mo	8.99 (7.89, 10.25)	0.84 (0.65, 1.08)	
4,501–7,500 Q/mo	5.82 (3.96, 8.55)	0.54 (0.35, 0.82)	

Indigenous, self-identified as being of indigenous ethnic background. Q, Guatemalan quetzales (1 USD ≈ 7.5 Q).

^*a*^ Values are pg AFB_1_-albumin / mg albumin.

^*b*^ Geometric means were simultaneously adjusted for age (continuous), sex, residence location, ethnicity, educational attainment category, and income category.

The rates of HBV and HCV infection in this sample were low. Positivity for HBsAg was found in only 4 of 439 participants (0.9%), while only 2 of 441 tested positive for anti-HCV (0.5%). Due to low prevalence, statistical analysis was not carried out for HBsAg or anti-HCV markers. Sixty-one of 441 participants (13.8%) tested positive for anti-HBc. The prevalence of anti-HBc positivity was associated with residence location (8.2% urban, 17.3% rural; p = 0.007) and with indigenous ethnicity status (17.2% yes, 10.0% no; p = 0.037). Males trended towards a higher proportion of anti-HBc positivity than females (17.6% *vs*. 11.0%, respectively; p = 0.051). Only 0.5% of individuals (2 / 439) tested positive simultaneously for HBsAg and anti-HBc.

## Discussion

In this study, we report the first examination of aflatoxin exposure in Guatemala using subject-level molecular biomarkers, alongside data showing low rates of HBV and HCV infection. Given the consumption of maize as a dietary staple throughout Central and South America, the affinity of *Aspergillus* species for colonization of maize, and the uncontrolled contamination of crops in much of the economically developing world [14], ingestion of aflatoxin-tainted food poses a threat of great damage to human health. While aflatoxin exposures in Mexico [31,32] and South America [33–36] have been documented with the use of subject-level biomarkers, no previous studies had examined exposures in Central American countries. Furthermore, given the fact that several nations in this region (Guatemala, El Salvador, Nicaragua) experience a highly unusual 1:1 ratio of LC incidence between men and women, studying these populations may reveal particular etiological insight. Finally, as LC in the United States is projected to rise most rapidly in the Hispanic community [37], knowledge of risk factors and disease patterns in Central America could be informative for a broader geographic region.

LC incidence is rising in the U.S., particularly among Hispanics [38,39]. While in part a result of HCV infection in the aging “baby-boomer” generation [6,12,40], LC is now largely driven by the growing contribution of metabolic disorders [12]. In particular, in a recent analysis, the constellation of obesity, non-alcoholic fatty liver disease, insulin resistance, diabetes, and metabolic syndrome accounted for nearly 40% of hepatocellular carcinoma incidence in U.S. Hispanics [12]. Not only were these metabolic disorders found to be the largest factors driving hepatocellular carcinoma incidence across all ethnicities, Hispanics experienced the highest population attributable fraction calculated for any of the ethnic groups examined [12]. Similarly, the metabolic perturbations of obesity and diabetes likely play a significant role in Guatemalan LC incidence. In the same sample as the current study, 30% of Guatemalan adults were found to be obese, 22% had diabetes, and 63% met criteria for metabolic syndrome [41]. Notably, these factors were more prevalent in women than in men, some by nearly three-fold (*e*.*g*., obesity, 41% *vs*. 15%, respectively). Although not tied to cancer incidence, this data suggests that obesity and its sequelae likely plays a significant role in Guatemalan LC burden and may contribute to its uncommon distribution between men and women.

Previous work has shown that any detectable aflatoxin exposure was associated with significantly increased risk of hepatocellular carcinoma [22,23]. Thus, the high prevalence of detectable AFB_1_ exposure in the current study (100%) suggests that aflatoxins may play a significant role in LC etiology in Guatemala. Moreover, since AFB_1_-lys dose reflects the formation of mutagenic AFB_1_-DNA adducts *in vivo* [42], greater magnitude of aflatoxin exposure leads to higher risk of liver carcinogenesis. Participants overall–and particularly those who were male, indigenous, and/or rural–had AFB_1_-lys adduct levels similar to regions in China which experienced endemic rates of LC into the 1980s, prior to the reductions in mortality attributable to decreased aflatoxin exposure [11]. Furthermore, the data reported in this study can be used to infer subject-level estimates of average daily AFB_1_ intake by interpolation of our AFB_1_-lys biomarker data with prior dose-response results from ^14^C-AFB_1_ micro-dosing [43,44], approximation of chronic exposure using AFB_1_-albumin adduct clearance kinetics [45], and correction for body weight. Estimates of average chronic AFB_1_ intake in this work ranged from 0.05–1,877 μg AFB_1_/day, with a median of 18.4 μg/d (quartile 1, 6.1 μg AFB_1_/day; quartile 3, 50.3 μg AFB_1_/day). These intake estimates are similar to quantitative food-based measurements of total daily AFB1_1_ intake in other regions with high rates of liver cancer [46,47].

Among the sub-groups in our analysis, rural and indigenous Guatemalans experienced the highest levels of aflatoxin exposure. Moreover, males in each of these demographic sub-groups experienced further elevated AFB_1_ exposures. Previous studies in other populations have found that AFB_1_ exposure was associated with male sex in Taiwanese adults [48], residence location in children in Benin and Togo [49] and Tanzania [50], and ethnicity in pregnant Bangladeshis [51]. Additionally, we found an interaction in AFB_1_-lys levels between two of these factors, where unadjusted circulating adducts were lower in non-indigenous urban *vs*. non-indigenous rural participants, but equal in indigenous participants, regardless of location of residence. This interaction suggests that cultural or other factors affect aflatoxin adduct levels, perhaps through food choice or post-harvest practices [14]. However, ethnic self-identification and location of residence were closely related in our sample–the majority of rural participants self-identified as being of indigenous ancestry (83.0%), while urban participants were predominantly non-indigenous (91.6%). After simultaneous adjustment for all demographic characteristics, residence location, but not self-identified indigenous status, was significantly associated with AFB_1_ exposure. Further work will be required to fully dissect the impacts of these factors on aflatoxin exposure in Guatemala.

In addition to aflatoxin exposure, we assessed the prevalence of infection by HBV and HCV, which, when chronic, are major etiological factors of primary liver cancer [8,28]. Chronic HBV infection is defined as persistent HBsAg seropositivity for at least 6 months, which typically occurs alongside a positive anti-HBc result [52]. Seropositivity for anti-HCV suggests chronic infection with HCV [53]. Seminal work has previously demonstrated a greater-than-multiplicative interaction between concurrent aflatoxin exposure and chronic HBV infection, with estimates of liver cancer risk being 60-fold greater than with neither exposure [22,23]. The apparent low rates of chronic HBV and HCV infection observed in our study are similar to a previous country-level report from Guatemala [54], lower than regional estimates in Latin America [55], and much lower than historically endemic rates in Asia [56]. A vaccine for HCV is not available and improved HBV vaccination coverage in Guatemala is still needed—WHO has estimated that, in 2015, only 32% of newborns in the country were vaccinated by 1 year of age [57]. Despite this, rates of chronic HBV and HCV infection in Guatemala appear to be low, even while the country suffers from high rates of hepatocellular carcinoma. These data stand in contrast to aflatoxin exposure in Guatemala, which we report to be widespread and of significant magnitude.

Strengths of the current study include the sampling of diverse communities and, in particular, the use of subject-level molecular biomarkers to precisely determine aflatoxin exposure and HBV and HCV infection. Limitations of our study are the cross-sectional design and that the sampling method was not designed to be nationally representative. However, our sample demographics are similar to national-level estimates. In conclusion, this is the first study to measure biomarkers of aflatoxin exposure in Guatemala, a country which suffers from LC rates triple that of the Americas region as a whole. We have identified a high prevalence of exposure to AFB_1_, with quantitative levels similar to those previously found to increase risk for LC. Simultaneously, low rates of HBV and HCV infection suggest that these viruses may not play a major etiological role in LC in Guatemala. Combined with the high prevalence of obesity and metabolic syndrome, Guatemala presents a unique profile of LC risk factors (aflatoxin exposure, low HBV and HCV, high rates of obesity and fatty liver disease) that are similar to Hispanic communities in the U.S. with LC or cirrhosis [58,59]. Thus, Guatemala may encapsulate what appears to be a new and growing paradigm of LC etiology in both developed and developing nations. Finally, these findings could be used to inform appropriate cancer prevention strategies in Guatemala–aflatoxin exposure is a modifiable risk factor and effective, scalable, culturally competent, and economically feasible prevention strategies have been proposed and validated in low- and middle-income countries [14]. Further work to examine the associations between LC risk factors and incidence in this population is clearly needed.
